# Cerebral edema in maple syrup urine disease: spectrum of clinical presentation and treatment outcomes

**DOI:** 10.1186/s13023-025-04043-1

**Published:** 2025-11-19

**Authors:** Raashda A. Sulaiman, Ruqaiah Altassan, Randa Alshammari, Moeenaldeen Alsayed, Aamir N. Sheikh, Manal Nicolas-Jilwan, Mohamed Al-Owain, Zuhair Al-Hassnan

**Affiliations:** 1 Department of Medical Genomics, MBC: 75, Genomic Medicine Center of Excellence, King Faisal Specialist Hospital and Research Center, PO Box No: 3354, 11211 Riyadh, Saudi Arabia; 2 Department of Medicine, Medical Center of Excellence, King Faisal Specialist Hospital and Research Center, Riyadh, Saudi Arabia; 3 Radiology Division, King Faisal Specialist Hospital and Research Center, Riyadh, Saudi Arabia; 4 College of Medicine, Alfaisal University, Riyadh, Saudi Arabia

**Keywords:** MSUD, Leucine, Encephalopathy, Cerebral edema, Papilledema

## Abstract

**Background:**

Maple syrup urine disease (MSUD) is an inherited neurometabolic disorder caused by a deficiency of branched-chain α-keto acid dehydrogenase complex activity. There is an accumulation of neurotoxic branched-chain amino acids and their corresponding alpha-ketoacids. During acute metabolic decompensation, there is a high risk of mortality due to encephalopathy and cerebral edema, leading to cerebellar herniation.

**Subjects and methods:**

This study reviewed the clinical presentation, management, and outcome of adult patients with MSUD who were admitted to our hospital with encephalopathy and cerebral edema during the 8-year study period.

**Results:**

Seven patients were admitted with ten episodes of encephalopathy, and cerebral edema was present during nine episodes. One asymptomatic patient had an elective admission with cerebral edema. Five patients had a full recovery to baseline, while two patients died.

**Conclusions:**

This study describes the variable clinical presentation of cerebral edema in adult patients with MSUD. Early recognition and prompt treatment of encephalopathy is challenging, particularly in adult patients, as the multidisciplinary teams may not be familiar with this rare disease.

## Introduction

Maple syrup urine disease (MSUD; OMIM 248600) is an autosomal recessive neurometabolic disorder caused by the deficiency of the mitochondrial branched-chain α-ketoacid dehydrogenase (BCKD) complex, which catalyzes the branched-chain α-ketoacid derivatives (BCKA) of branched-chain amino acids (BCAA). Biallelic loss of function mutations in one of the three genes (*BCKDHA, BCKDHB,* and *DTB*) that encode BCKD subunits result in the accumulation of neurotoxic BCAA (leucine, isoleucine and valine) and their corresponding BCKA [1]. The worldwide incidence of MSUD is ~ 1:185,000. However, certain consanguineous populations have a much higher incidence (1:400 in the Old-Order Mennonites in the United States and 1:15,816 in screened newborn babies in Saudi Arabia) [2, 3].

An acute increase in leucine and BCKA during intercurrent illness, prolonged fasting or poor dietary compliance causes encephalopathy, which manifests as a spectrum ranging from irritability, confusion, and hallucinations to seizures, altered level of consciousness and coma [4, 5]. Patients with encephalopathy may remain fully conscious in early stages, scoring high on the Glasgow Coma Scale (GCS).

The exact pathophysiology of cerebral edema in MSUD is not entirely understood. Leucine competes with other amino acids (glutamine, glutamate, phenylalanine, tyrosine, tryptophan, and methionine) to enter the brain via the *SLCA45*-encoded transporter. High leucine concentration reduces the cerebral essential amino acids, leading to neurotransmitter deficiency [4–7]. It also reduces the Na^+^ and K^+^-ATPase activity and disrupts cerebral water homeostasis, precipitating cerebral edema during acute crises [8, 9]. Excessive accumulation of BCAAs and BCKAs in the brain disrupts redox homeostasis, impairs mitochondrial bioenergetics by disturbing the Krebs cycle, leading to energy deprivation and increased cerebral lactate concentration, particularly during metabolic crises [4, 6, 10]. There is a positive correlation between pro-inflammatory cytokines in the brain (interleukin 6, interleukin 1β) and the frequency of metabolic crises [11–13].

Most of the literature and reported experiences of metabolic decompensation in MSUD are about pediatric patients. This is a retrospective study of MSUD patients aged ≥ 14 years who were admitted to this tertiary care facility with encephalopathy during the 8-year study period. It elaborates on the spectrum of clinical manifestations, the management and outcomes of cerebral edema.

## Subjects and methods

This retrospective study was conducted by reviewing the medical records of patients with MSUD who were admitted with encephalopathy during the study period from 2016 to 2023. Data on their acute presentation, laboratory results, imaging, treatment, and clinical outcomes were collected.

This study was approved by the hospital research advisory council (RAC # 2221255) and conducted in compliance with the hospital's ethical standards and guidelines.

## Results

Seven patients (6 males and 1 female) presented with ten episodes of acute encephalopathy, and cerebral edema was present during nine episodes. One asymptomatic patient was readmitted electively as cerebral edema was noted on an outpatient MRI of the brain.

All patients had classic form of MSUD, diagnosed in the neonatal period by plasma amino acid analysis, when they were admitted with encephalopathy. The diagnosis was later confirmed by genetic analysis. Their demographic data, genetic mutations, and clinical, biochemical, and imaging profiles on admission are shown in the Table 1.. They had infrequent routine plasma amino acid monitoring. Baseline plasma leucine levels were much higher in patients with poor dietary compliance (Table 1.).

**Table 1:** Clinical, molecular data with biochemical and imaging profile of the patients with MSUD on admission

	Patient 1	Patient 2	Patient 3	Patient 4	Patient 5	Patient 6	Patient 7
Genetic mutation (Homozygous)	DBT c.360del p.Lys120AsnfsTer6	BCKDHB c.840+2T>G	BCKDHA c.196G>A p.Gly66Arg	DBT c.30G>A p.Trp10^*^	DBT c.30G>A p.Trp10^*^	DBT c.1274C>G p.Ser425^*^	BCKDHB c.347A>G p.Asp116Gly
Height (cm)	168	147	149.5	141	167	145	160
Weight (Kg)	53	46	40	67	49	35	40
Neuro psychiatric deficit	ADHD	Speech deficit	-	-	-	GDD, spastic tetraplegia	GDD, spastic tetraplegia
Intellectual disability	Mild	Moderate	Mild	Moderate	Moderate	Severe	Severe
Seizure	Absent	Present	Absent	Absent	Absent	Present	Present
Dietary & special formula compliance	Poor	Poor	Satisfactory	Not taking formula lately	Not taking formula lately	Satisfactory	Satisfactory
Base line leucine levels(N<200 µmol/L)	800-1200	600-1000	300-600	200-300	200-600	200-400	200-350
Number of admissions	Three	Two (2^nd^elective)	One	One	Two	One	One
Plasma leucine (N<200 µmol/L	1^st^ adm. 1938 2^nd^ adm. 1363 3^rd^ adm. 2090	1^st^ adm 1290 2^nd^ adm 1330	2400	2030	1^st^ adm. 1425 2^nd^ adm. 1366	1206	2301
Plasma isoleucine (N<120 µmol/L)	1^st^ adm. 550 2^nd^ adm. 222 3^rd^ adm. 314	1^st^ adm. 320 2^nd^ adm. 362	1465	516	1^st^ adm. 634 2^nd^ adm. 376	297	533
Plasma valine (N<300µmol/L)	1^st^ adm. 829 2^nd^ adm. 288 3^rd^ adm. 752	1^st^ adm. 643 2^nd^ adm. 471	1131	1018	1^st^ adm. 886 2^nd^ adm. 668	318	1210
Cerebral edema on imaging	1^st^adm. Present 2^nd^ adm. Present 3^rd^ adm. Present	1^st^adm. - 2^nd^adm. Present	Present	Present	1^st^adm. Present 2^nd^adm. Present	Present	Present
Clinical outcome	Recovered	Recovered	Died	Died	Recovered	Recovered	Recovered

ADHD: attention deficit hyperactivity disorder; GDD: global developmental delay; adm: admission

**Fig. 1:**
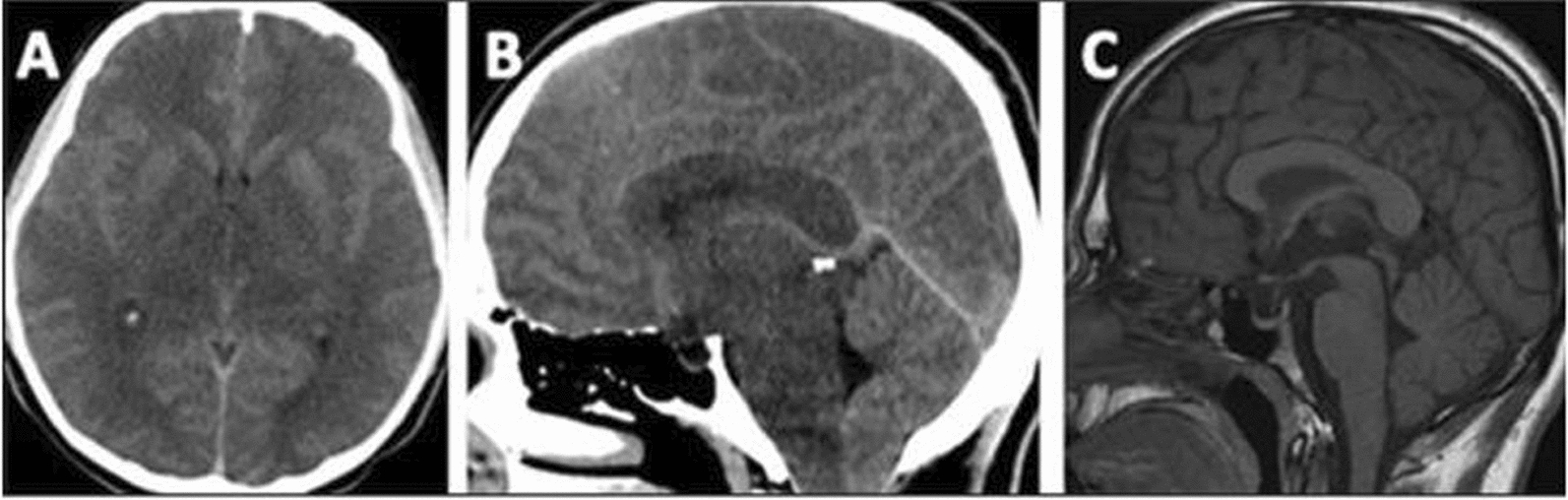
Brain imaging of patient 1 Head-CT at the initial presentation shows diffuse brain edema (**A** and** B**), causing cerebellar tonsillar herniation (**B**). Sagittal T1 from the following MRI brain shows improved edema and resolution of tonsillar herniation (**C**)

Respiratory tract infection and poor dietary compliance were the trigger factors for acute metabolic crises in most patients. The hospital course of these patients during acute illness is summarized as follows:

**Patient 1**: A 21-year-old male patient presented with nausea, vomiting, and reduced oral intake for 3 days. He looked lethargic, although fully conscious, with a GCS of 15/15. Due to poor dietary compliance, plasma leucine levels were chronically elevated (1300–1600 µmol/L) for 1 year prior to this episode. He was admitted as a case of metabolic decompensation and received conservative treatment with IV dextrose in saline infusion, BCAA-free formula and dietary natural protein restriction. The next day of admission, he had a headache and transient abnormal hand movements. A CT scan of the brain showed diffuse cerebral edema causing cerebellar tonsillar herniation (Fig. 1: A and B). He remained fully conscious with no focal neurological signs. Plasma leucine was 1300, isoleucine 237 and valine 271 µmol/L. He was shifted to the ICU and received continuous renal replacement therapy (CRRT) for 48 h until leucine levels were reduced to 400 µmol/L. Conservative management continued with hypertonic saline infusion, high-calorie intake, BCAA-free formula and valine and isoleucine supplements. He remained clinically stable and asymptomatic; however, a repeat CT scan after 5 days showed persistent diffuse cerebral edema and cerebellar tonsillar herniation.

Following discharge from the hospital, the patient developed bilateral visual impairment. His leucine levels were satisfactory (200 µmol/L). Ophthalmological examination revealed severe bilateral papilledema with a visual acuity of 20/200 in the right eye and 20/300 in the left eye. A repeat CT scan showed improvement in cerebral edema with persistent but improved cerebellar tonsillar herniation. He was prescribed acetazolamide for papilledema. Plasma leucine levels were 200–600 µmol/L in the following few months. CT brain scan after 12 weeks of the initial presentation showed further improvement of cerebral edema and resolution of cerebellar tonsillar herniation. However, his vision remained poor, which justified magnetic resonance imaging (MRI; Fig. 1C) demonstrating persistent brain edema in a distribution compatible with MSUD extending to the optic tracts. The optic nerves were mildly atrophic, with a mildly abnormal signal in the optic chiasma. His dietary compliance, which initially improved for a few months after discharge from the hospital, gradually worsened.

During the study period, he had two more admissions, one 3 years prior to this admission and another, 2 years following this admission, when he presented with nausea, vomiting, and a poor appetite. He was fully conscious with a GCS of 15/15 and was noted to have diffuse cerebral edema on CT scan on both occasions. During those admissions, he received renal replacement therapy in addition to conservative management, recovered clinically and was discharged.

**Patient 2**: A 19-year-old female was admitted with epigastric pain, nausea, visual hallucinations, and ataxia. Her BCAAs were high (Table). She also had acute pancreatitis as the plasma lipase was 620 IU/L (< 60). She fully recovered following conservative management. After discharge, she remained non-compliant with the protein-restricted diet. Her amino acid profile consistently showed high leucine levels (1200–1700 µmol/L) for several months, although she remained asymptomatic. An outpatient brain MRI revealed vasogenic edema in the bilateral basal ganglia, thalami, and deep white matter extending into the brainstem and cerebellar hemispheres. Her neurological examination was unremarkable. Plasma BCAA levels were high (Table 1.). She was admitted for conservative management and was discharged after four days when her leucine level improved to 600 µmol/L. An ophthalmological examination ruled out papilledema. Her follow-up BCAA levels remained in the higher range.

**Patient 3:** A 14-year-old male presented to the emergency room (ER) with a history of fever and reduced activity. He appeared well clinically and had unremarkable routine blood test results. He was discharged on antipyretics. The next day, he presented with a reduced level of consciousness, a GCS of 9/15 and had an episode of seizure in the ER. CT brain was unremarkable. BCAA on admission were high (Table 1.). In addition to IV fluids, BCAA-free formula, CRRT was started. Initially, there were frequent and prolonged interruptions in CRRT due to technical issues, and the leucine level was 2058 µmol/L after 48 h. There was no improvement in the level of consciousness. A repeat CT scan on the third day showed diffuse cerebral edema. CRRT continued with hypertonic saline infusion. Plasma leucine levels were reduced to 560 µmol/L when he developed fixed dilated pupils and loss of gag reflex, with confirmation of cerebellar tonsillar herniation on a CT scan. He died on the 15th day of admission.

**Patient 4:** A 15-year-old male patient presented with agitation, unsteady gait, and visual hallucinations, seeing "snakes on the wall" after a flu-like illness and recurrent vomiting for 3 days. His GCS was 15/15 on admission. He had not taken the BCAA-free formula for a few months. BCAA levels were high on admission (Table 1.) and CT brain showed diffuse edema. He received IV fluids, BCAA-free formula, and CRRT was initiated. He later became drowsy and required intubation and ventilatory support. On the third day, when plasma leucine was reduced to 1033 µmol/L, he developed sinus tachycardia and hypertension. He had fixed dilated pupils, with no corneal or gag reflex. CT- brain confirmed the worsening of cerebral edema with cerebellar tonsillar herniation. He underwent urgent bifrontal decompressive craniotomy but died on the 9th day of admission.

**Patient 5:** A 16-year-old male, the elder brother of patient 4, presented with fever and vomiting for 4 days. He was irritable and had visual hallucinations. GCS was 15/15 on admission. Plasma BCAA levels were high (Table 1.). The CT brain showed diffuse cerebral edema. He received IV fluids, BCAA-free formula and underwent hemodialysis twice with a resolution of acute symptoms. He was readmitted after two years with flu-like symptoms, irritability, headache, photophobia, visual hallucination, and unsteady gait. He received IV fluids, BCAA-free formula, and CRRT was initiated. Plasma leucine levels were reduced to 616 µmol/L in 48 h with marked clinical improvement. He was discharged in 9 days. There were no neurological sequelae.

**Patient 6:** A 16-year-old male presented with fever, vomiting, and confusion. His GCS was 9/15. He had an upper respiratory tract infection. The initial BCAA levels were high (Table 1.), and the CT brain showed cerebral edema. He responded well to antibiotics and conservative management, fully recovering to his baseline, and was discharged in 8 days.

**Patient 7:** A 28-year-old male patient with seizure disorder, spastic quadriplegia, erosive esophagitis and G-tube feeding, was admitted with vomiting, fever and drowsiness (GCS,7/15). He had aspiration pneumonia. He received antibiotics, IV fluids, and a BCAA-free formula; CRRT was initiated. Plasma leucine was reduced to 400 µmol/L with clinical improvement to baseline status, and the patient was discharged in 12 days. He was readmitted after 6 months in a state of shock, following severe vomiting and diarrhea. He was drowsy, febrile, severely dehydrated, and chest X-ray showed collapse consolidation in the left lobe. He did not respond to conservative treatment and died.

## Discussion

Encephalopathy and cerebral edema are life-threatening complications of MSUD. This case series demonstrates a broad spectrum of encephalopathy related to MSUD and reports the unusual presentation of cerebral edema in adult patients with MSUD. Of all 10 admissions with cerebral edema, during the four admissions, patients (1, 2) remained fully conscious with no neurological signs or symptoms. Two other patients (4, 5) with cerebral oedema noted on admission were also fully conscious, ambulating and communicating, although irritable, and had hallucinations.

Patient 1, with extensive cerebral edema and cerebellar tonsillar herniation seen on a CT scan, was not suspected of having encephalopathy or cerebral edema on admission and was initially managed as a case of metabolic decompensation. He later developed severe visual impairment after a marked reduction in plasma leucine levels and improvement in cerebral edema. Papilledema and MRI findings, including the extension of cerebral edema to the bilateral optic pathway and optic nerve atrophy, explained his visual impairment. Papilledema with visual impairment is a known presenting feature of acute metabolic decompensation [14, 15]. It is unusual that he developed visual impairment during recovery with much reduced plasma leucine levels, possibly due to the development of optic nerve atrophy at that stage. This case highlights the importance of a comprehensive eye examination, including fundoscopy, as a mandatory part of the acute assessment of these patients. Furthermore, Optical Coherence Tomography (OCT), an invaluable tool for quantitatively assessing optic nerve edema and subsequent atrophy, is highly recommended as a standard of care for evaluating and monitoring potential optic nerve damage, particularly in patients with persistent edema or chronic hyperleucinemia.

MSUD edema primarily involves the cerebellar white matter, brainstem, globus pallidus, internal capsule, and thalamus [16, 17]. Although CT scan is readily available in an emergency, it shows generalized edema while MRI brain shows detailed MSUD specific changes, especially when using diffusion-weighted imaging (DWI), which can distinguish between intramyelinic edema and vasogenic edema. MRI is therefore, highly recommended as the initial imaging modality of choice for diagnosing and monitoring cerebral edema in MSUD and detecting early ischemic changes in vulnerable areas like the brainstem, cerebellum, and optic nerve.

Patients 1 and 2 demonstrate a critical disparity that may exist between clinical appearance and the severity of underlying cerebral pathology in adult patients**.** Chronic progressive hyperleucinemia for several months may explain the insidious, silent development of cerebral edema and cerebellar herniation. The cerebral edema takes longer to resolve, even when plasma leucine levels remain low. These patients should be closely monitored for further neurological complications.

Dietary compliance is quite demanding in adult MSUD patients with psychosocial issues and learning disability. Adult patients are known to have higher BCAA levels and more frequent metabolic decompensation than pediatric patients [18].

There are critical gaps in knowledge on fully understanding cerebral pathophysiology during an acute crisis. Plasma leucine levels do not correlate with cerebral leucine levels and the degree of cerebral edema [4, 19]. Patients developed acute encephalopathy and cerebral edema when plasma leucine levels improved with dietary management [20–22]. We lost two patients with severe cerebral edema who developed cerebellar herniation and became unresponsive with dilated nonreactive pupils and loss of gag reflex despite improvement in plasma leucine levels. This highlighted the need for an advanced, noninvasive intracranial pressure monitoring technique to prevent fatal outcomes in such cases.

Early recognition and management of acute metabolic decompensation are specifically challenging in adult patients. Frontline ER physicians may not recognize the initial subtle signs and symptoms. In the presence of unremarkable routine blood tests, these patients may not be admitted, as happened in patient 3. Early nonspecific symptoms such as poor appetite, nausea, vomiting, lethargy, and headache may not be recognized as a warning for impending metabolic crises. Patients may present with hallucinations, aggressive behavior, and confusion [4, 23, 24]. These symptoms can rapidly progress to an altered level of consciousness, seizures, coma, and death. A high suspicion index is required for the early recognition of encephalopathy in these patients, as plasma amino acid analysis may not be available urgently, and other biochemical test results may remain unremarkable. Treatment should not be delayed for any pending amino acid analysis.

The goal of acute management during metabolic decompensation is to suppress catabolism and promote protein anabolism. We have developed an inpatient protocol to facilitate the effective multidisciplinary management of MSUD crises in adult patients. It is similar to the acute management plan described in the consensus guidelines on the diagnosis and management of MSUD [25]. Dextrose 10–12.5% in normal saline infusion, with or without lipid infusion, provides 1.5 times the estimated energy expenditure (EER) and is carefully adjusted to maintain serum sodium at 138–145 mmol/L and plasma osmolality at 285–300 mOsm/kg H_2_O. During acute crises, hyponatremia may develop due to renal sodium loss [20], and dextrose infusion may worsen cerebral edema, making fluid management exceptionally challenging. Blood glucose is monitored regularly, and if significantly elevated, it is corrected by insulin infusion. Dietary natural protein is withheld for 12–48 h; a BCAA-free amino acid formula is administered to achieve a total protein equivalent intake of 2.0–3.5 g/kg/day via oral or nasogastric route. Valine and isoleucine supplements (20–120 mg/kg/day) are given via the enteral route to promote protein synthesis and reduce leucine levels. Furosemide, mannitol, and hypertonic saline administration should be considered in patients with cerebral edema. Hemodialysis and CRRT have been successfully used in managing MSUD crises in pediatric patients [26–28]. However, there is no publication in the literature on the experience of such therapy in adult patients with MSUD. Our experience shows that early renal replacement therapy can be effective for the rapid reduction in plasma leucine levels in adults with severe encephalopathy and brain edema.

The treatment should be supervised by a metabolic physician in close collaboration with other services. However, the hospital may not have an adult metabolic physician; adult intensive care unit, and other adult specialties may not be familiar with managing acute crises in MSUD. All these factors contribute to a precarious and challenging situation, emphasizing the need for close collaboration among all services and for extremely vigilant monitoring of patients to achieve better clinical outcomes. 

## Conclusion

This study describes the spectrum of clinical presentation of cerebral edema in patients with MSUD, ranging from asymptomatic to a severely encephalopathic state. It highlights the insidious and life threatening nature of cerebral edema that may develop in adult patients with chronic progressive hyperleucinemia. Early recognition and prompt treatment are crucial and challenging, particularly in adult patients, as the multidisciplinary teams may not be familiar with the effective management of this rare disease.

## Data Availability

Data are stored on a secure hospital server with no external access. Before sharing any data, we need to get the relevant permission.

## References

[1] Schiff M, de Baulny HO, Dionisi-Vici C. Branched-chain organic acidurias/acidaemias. In: Saudubray JM, Baumgartner MR, Walter J, editors. Inborn metabolic diseases: diagnosis and treatment. 6th ed. Heidelberg: Springer-Verlag; 2016. p. 279–88.

[2] Chapman KA, Gramer G, Viall S, et al. Incidence of maple syrup urine disease, propionic acidemia, and methylmalonic aciduria from newborn screening data. Mol Genet Metab Rep. 2018;15:106–9. 30023298 10.1016/j.ymgmr.2018.03.011 PMC6047110

[3] Alfadhel M, Al Othaim A, Al Saif S, et al. Expanded newborn screening program in Saudi Arabia: incidence of screened disorders. J Paediatr Child Health. 2017;53(6):585–91. 28337809 10.1111/jpc.13469

[4] Strauss KA, Carson VJ, Soltys K, et al. Branched-chain α-ketoacid dehydrogenase deficiency (maple syrup urine disease): treatment, biomarkers, and outcomes. Mol Genet Metab. 2020;129(3):193–206. 31980395 10.1016/j.ymgme.2020.01.006

[5] Strauss KA, Wardley B, Robinson D, et al. Classical maple syrup urine disease and brain development: principles of management and formula design. Mol Genet Metab. 2010;99:333–45. 20061171 10.1016/j.ymgme.2009.12.007 PMC3671925

[6] Zinnanti WJ, Lazovic J, Griffin K, et al. Dual mechanism of brain injury and novel treatment strategy in maple syrup urine disease. Brain. 2009;132:903–18. 19293241 10.1093/brain/awp024 PMC2668944

[7] Xu J, Jakher Y, Ahrens-Nicklas RC. Brain branched-chain amino acids in Maple Syrup Urine Disease: implications for neurological disorders. Int J Mol Sci. 2020;21(20):7490. 33050626 10.3390/ijms21207490 PMC7590055

[8] Rosa L, Galant LS, Dall’Igna DM, et al. Cerebral oedema, blood-brain barrier breakdown and the decrease in Na (+), K(+)-ATPase activity in the cerebral cortex and hippocampus are prevented by dexamethasone in an animal model of maple syrup urine disease. Mol Neurobiol. 2016;53:3714–23. 26133302 10.1007/s12035-015-9313-0

[9] Blackburn PR, Gass JM, Vairo FPE, et al. Maple syrup urine disease: mechanisms and management. Appl Clin Genet. 2017;10:57–66. 28919799 10.2147/TACG.S125962 PMC5593394

[10] Amaral AU, Wajner M. Pathophysiology of maple syrup urine disease: focus on the neurotoxic role of the accumulated branched-chain amino acids and branched-chain α-keto acids. Neurochem Int. 2022;157:105360. 35577033 10.1016/j.neuint.2022.105360

[11] Scaini G, Tonon T, Moura de Souza CF, et al. Evaluation of plasma biomarkers of inflammation in patients with maple syrup urine disease. JIMD. 2018;41:631–40. 10.1007/s10545-018-0188-x 29740775

[12] Wessler LB, Ramos De Miranda, Pasquali B, et al. Administration of branched-chain amino acids increases the susceptibility to lipopolysaccharide-induced inflammation in young Wistar rats. Int J Dev Neurosci. 2019;78:210–4. 31330240 10.1016/j.ijdevneu.2019.07.007

[13] Rabelo F, Lemos IDS, Dal Toé C, et al. Acute effects of intracerebroventricular administration of α-ketoisocaproic acid in young rats on inflammatory parameters. Metab Brain Dis. 2023;38(5):1573–9. 36897514 10.1007/s11011-023-01193-8

[14] Culleton S, Siddiqui A. Maple syrup urine disease decompensation presenting as papilloedema. Can J Neurol Sci. 2019;46(6):780–1. 31387654 10.1017/cjn.2019.253

[15] Sutter R, Killer HE, Bilz S, et al. Cerebral oedema and intracranial hypertension in an adult with maple syrup urine disease. Eur J Neurol. 2009;16(3):45–6. 10.1111/j.1468-1331.2008.02425.x 19364339

[16] Cheng A, Han L, Feng Y, Li H, et al. MRI and clinical features of maple syrup urine disease: preliminary results in 10 cases. Diagn Interv Radiol. 2017;23(5):398–402. 28830848 10.5152/dir.2017.16466 PMC5602367

[17] Ha JS, Kim TK, Eun BL, et al. Maple syrup urine disease encephalopathy: a follow-up study in the acute stage using diffusion-weighted MRI. Pediatr Radiol. 2004;34:163–6. 14504844 10.1007/s00247-003-1058-7

[18] Abi-Wardé MT, Arnoux JB, Servais A, et al. Long-term metabolic follow-up and clinical outcome of 35 patients with maple syrup urine disease. J Inherit Metab Dis. 2017;40(6):783–92. 28905140 10.1007/s10545-017-0083-x

[19] Vogel KR, Arning E, Wasek BL, et al. Brain-blood amino acid correlates following protein restriction in murine maple syrup urine disease. Orphanet J Rare Dis. 2014;9:73. 24886632 10.1186/1750-1172-9-73 PMC4022424

[20] Sen K, Gropman A, Harrar D. In-hospital mortality from cerebral edema in MSUD during newborn screening era: what are we missing and what more can we do? Pediatr Neurol. 2022;135:61–2. 36027849 10.1016/j.pediatrneurol.2022.07.013

[21] Myers KA, Reeves M, Wei XC, et al. Cerebral edema in maple syrup urine disease despite newborn screening diagnosis and early initiation of treatment. JIMD Rep. 2012;3:103–6. 23430881 10.1007/8904_2011_69 PMC3509865

[22] Brismar J, Aqeel A, Brismar G, et al. Maple syrup urine disease: findings on CT and MR scans of the brain in 10 infants. AJNR. 1990;11(6):1219–28. 2124065 PMC8332126

[23] Sulaiman RA, Alali A, Hosaini S, et al. Emergency management of critically ill adult patients with inherited metabolic disorders. Am J Emerg Med. 2022;55:138–42. 35313229 10.1016/j.ajem.2022.02.053

[24] Hashimoto T, Whitehead MT, MacLeod E, et al. Maple syrup urine disease decompensation is misdiagnosed as a psychotic event. Mol Genet Metab Rep. 2022;18:32. 10.1016/j.ymgmr.2022.100886 PMC9218201 35756860

[25] Rostampour N, Dalili S, Moravej H, et al. Comprehensive Iranian guidelines for the diagnosis and management of maple syrup urine disease: an evidence- and consensus- based approach. Orphanet J Rare Dis. 2025;20(1):8. 39773751 10.1186/s13023-025-03533-6 PMC11707931

[26] Puliyanda DP, Harmon WE, Peterschmitt MJ, et al. Utility of hemodialysis in maple syrup urine disease. Pediatr Nephrol. 2002;17(4):239–42. 11956873 10.1007/s00467-001-0801-2

[27] Atwal PS, Macmurdo C, Grimm PC. Haemodialysis is an effective treatment in acute metabolic decompensation of maple syrup urine disease. Mol Genet Metab Rep. 2015;4:46–8. 26937409 10.1016/j.ymgmr.2015.07.001 PMC4750565

[28] Deger I, Çelik M, Taş I, et al. Continuous veno-venous hemodiafiltration in neonates with maple syrup urine disease. Ther Apher Dial. 2022;26(3):658–66. 35166449 10.1111/1744-9987.13816

